# *Microchiritahairulii* (Gesneriaceae), a new species from Perlis, Peninsular Malaysia

**DOI:** 10.3897/phytokeys.118.32186

**Published:** 2019-03-01

**Authors:** Rafidah Abdul Rahman

**Affiliations:** 1 Forest Research Institute Malaysia, 52109 Kepong, Selangor, Malaysia Forest Research Institute Malaysia Selangor Malaysia

**Keywords:** conservation, flora, limestone, Malaysia, taxonomy

## Abstract

A new species, *Microchiritahairulii* Rafidah (Gesneriaceae) from limestone hills in Perlis, Peninsular Malaysia, is described and illustrated. Diagnostic characters, description, detailed illustrations, geographical distribution, regional provisional conservation status assessment (Endangered) and ecological observations of the new taxon, as well as an updated key to *Microchirita* species in Peninsular Malaysia, are provided.

## Introduction

In Peninsular Malaysia, *Microchirita* (C.B.Clarke) Yin Z.Wang comprises six species [viz., *M.involucrata* (Craib) Yin Z.Wang, *M.rupestris* (Ridl.) A.Weber & Rafidah and *M.viola* (Ridl.) A.Weber & Rafidah] with three endemics, *M.caliginosa* (C.B.Clarke) Yin Z.Wang, *M.ruthiae* Rafidah and *M.sericea* (Ridl.) A.Weber & Rafidah (Rafidah 2017). *Microchirita* grows exclusively in limestone habitats and its species are also found in India, Myanmar, southern China, Thailand, Vietnam, Laos, Cambodia, Sumatra, Java and Borneo, with Thailand as the centre of biodiversity with 28 species (Puglisi and Middleton 2017). Whilst conducting botanical exploration of limestone hills in Perlis under the limestone flora project for Peninsular Malaysia, I discovered an interesting *Microchirita* species on several limestone karst hills. The new species shows affinities with *M.caliginosa*, *M.sericea* in having a branched stem, pale purple corolla and hairy capsule. The new species also shows some similarities with *M.viola*. However, it is significantly different in the leaf shape, floral characters (flower size, indumentum of anthers) and seed characters (Table 1). To confirm that this taxon is indeed a new species, herbarium specimens and spirit material were taken back to the Forest Research Institute Malaysia herbarium (KEP), and living materials collected and grown in the nursery of FRIM were examined.

**Table 1.** Comparison of *Microchiritahairulii* with *M.caliginosa*, *M.sericea* and *M.viola*.

Characters	* M. hairulii *	* M. caliginosa *	* M. sericea *	* M. viola *
Stem	branched	branched	branched	branched
Lamina				
Arrangement	lowermost solitary opposite decussate	lowermost solitary opposite decussate	opposite	lowermost solitary opposite decussate
Shape	ovate	narrowly elliptic or elliptic	narrowly elliptic or narrowly ovate	ovate, sometimes orbicular
length (cm)	3.5–5.5(9)	6–15.5	4–11	3–8.5
width (cm)	2–2.2(6.2)	2.5–7	4–4.5	2–5.5
Base	cordate, cuneate, sometimes unequal	narrowly cuneate	attenuate, cordate	slightly cordate or rounded
Margin	serrate	serrulate	serrulate	serrate
Lateral vein pairs	5–6	5–10	5–10	6–16
Inflorescences	1–4-flowered	1–6-flowered	1–6-flowered	1–6-flowered
Corolla				
lobe colour	pale purple	pale purple	pale purple, lilac	violet
lobes stripes	faint	faint	conspicuous	conspicuous
tube length (mm)	5–10	24–55	10–26	20–22
Filaments	straight	slightly curved	slightly geniculate proximally	slightly geniculate
Anthers	free or connate	connate	connate	connate
Indumentum	glabrous	hairy	hairy	glabrous
Capsule				
length (mm)	15–18	20–80	15–70	*c.* 50
width (mm)	1–2	1.3–1.8	1.8–3	1–2
Indumentum	densely hairy	sparsely hairy	densely hairy	sparsely hairy
Seed				
surface	not papillate or canaliculate	papillate or canaliculate	papillate or canaliculate	rounded papillate

### Updated key to *Microchirita* species of Peninsular Malaysia

**Table:** 

1	Lamina narrowly elliptic or elliptic or narrowly ovate (sometimes in *M.sericea*) with widest point at the middle of the lamina	**2**
–	Lamina ovate, obovate or lanceolate with widest point below or above the middle of the lamina	**4**
2	Margin serrate. Corolla white, without conspicuous stripes	*** M. ruthiae ***
–	Margin serrulate. Corolla pale purple, with conspicuous stripes	**3**
3	Stem erect; lower leaf surface pale green to yellowish green, sometimes reddish green, base narrowly cuneate	*** M. caliginosa ***
–	Stem creeping; lower leaf surface very pale green or whitish green, base attenuate or sometimes cordate	*** M. sericea ***
4	Inflorescences pedunculate, bracts present	**5**
–	Inflorescences epiphyllous and crested, bracts absent (or minute)	**6**
5	Bracts connate-perfoliate into a cup-like arrangement	*** M. rupestris ***
–	Bracts not fused at base, leaf-like	*** M. involucrata ***
6	Calyx lobes 7–10 mm long, narrowly ovate. Corolla tube pale violet, lobes with conspicuous dark purple stripes; glandular hairs golden yellow, apically swollen in a cluster above the anther	*** M. viola ***
–	Calyx lobes 3–4 mm long, narrowly lanceolate. Corolla tube white or cream, pale yellow or pale purple, lobes very faintly striped or plain; with glandular hairs above the anthers, translucent pale brown	*** M. hairulii ***

## Taxonomy

### Microchirita hairulii

Taxon classificationPlantaeLamialesGesneriaceae

Rafidah sp. nov.

urn:lsid:ipni.org:names:77195469-1

**Figure 1.**
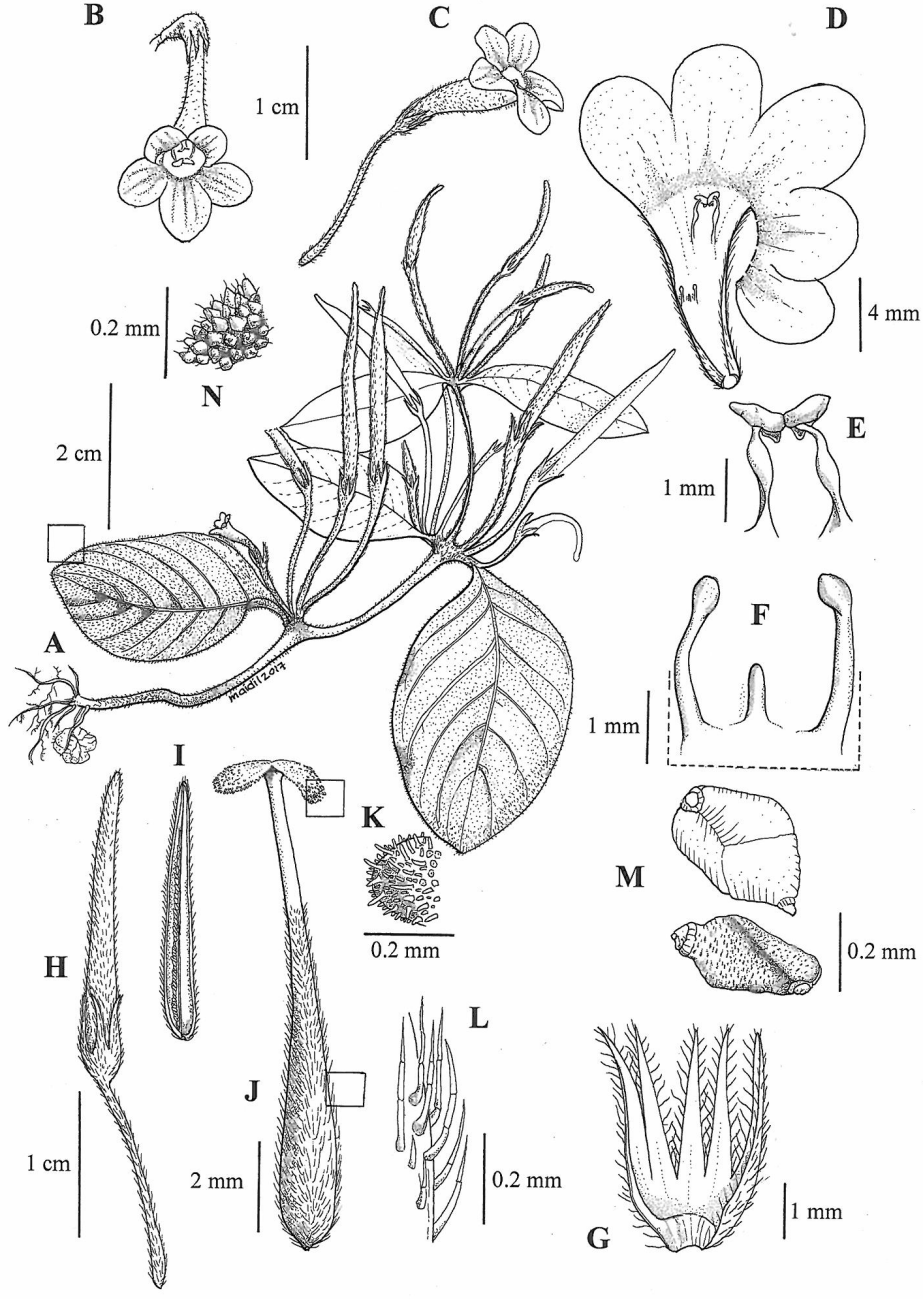
*Microchiritahairulii* Rafidah. **A** Habit **B** mature flower, front view **C** flower, side view **D** dissected corolla tube showing five lobes and a pair of stamens and staminodes **E** stamens **F** staminodes **G** calyx **H** fruit **I** LS section of fruit **J** pistil **K** indumentum of stigma **L** indumentum of ovary **M** seeds, upper and lower view **N** leaf epidermis with indumentum (*Rafidah FRI86669*). Drawn by Mohamad Aidil Noordin.

**Figure 2.**
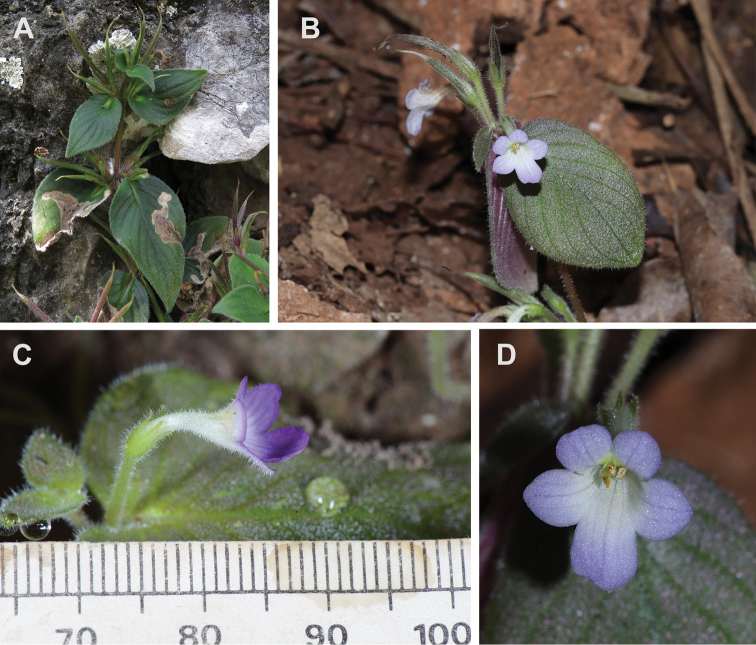
*Microchiritahairulii* Rafidah. **A** Habit **B** flowering and fruiting plant **C** flower, side view **D** flower, front view. Photographs **A, C, D** by Ong Poh Teck. Scale bar: 5 mm.

**Figure 3.**
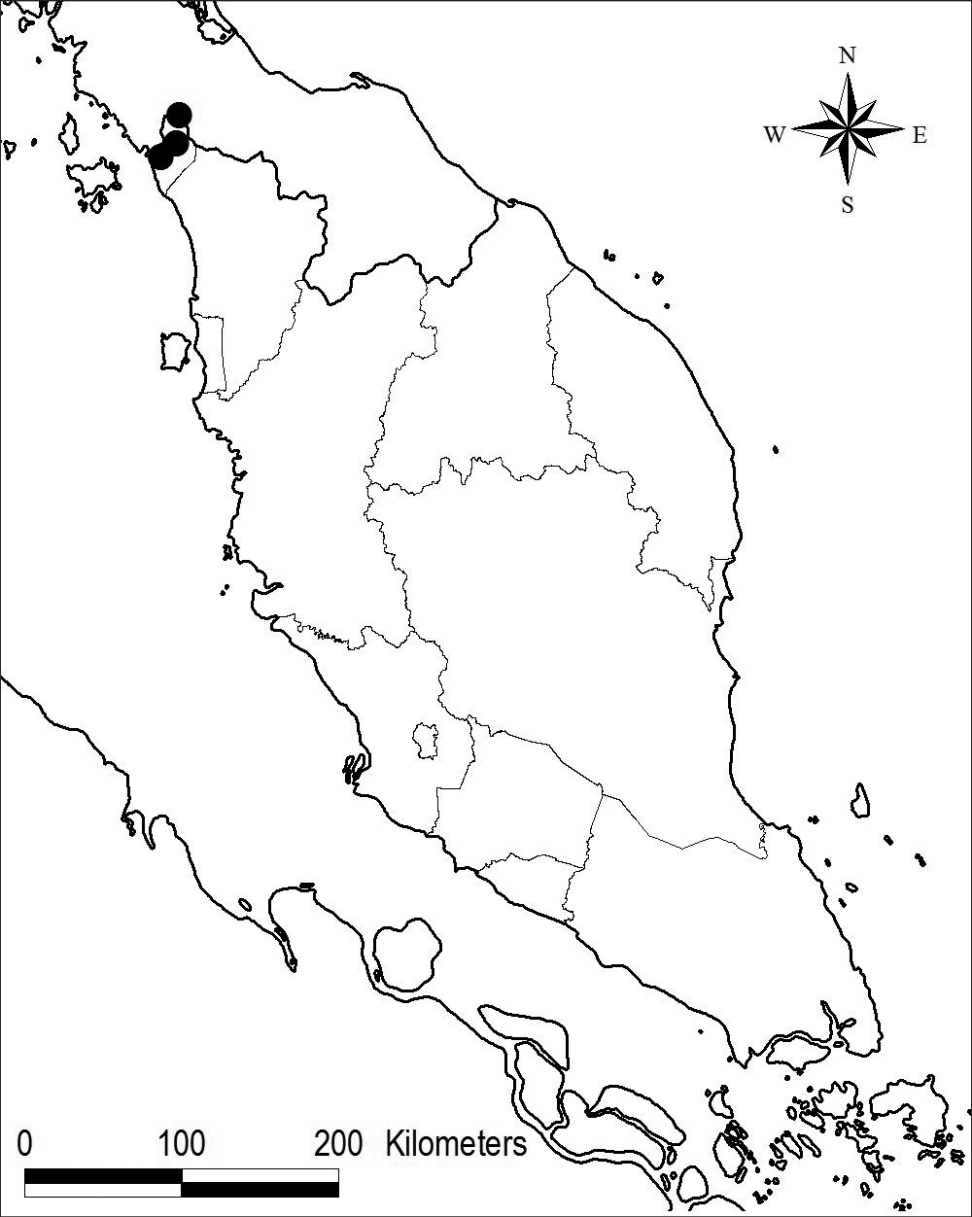
Distribution of *Microchiritahairulii* in Peninsular Malaysia.

Figures 1 , 2 , 3 , Table 1

#### Type.

Peninsular Malaysia, Perlis, Bukit Manik, 9 February 2017, *Rafidah FRI 86669* (holotype: KEP).

#### Diagnosis.

*Microchiritahairulii* most closely resembles *M.caliginosa* and *M.sericea* in having a branched stem, pale purple corolla and hairy capsule. This new species differs in having ovate leaves (vs elliptic to narrowly elliptic in *M.caliginosa* and *M.sericea* or sometimes narrowly ovate in *M.sericea*), serrate leaf margin (vs serrulate in *M.caliginosa* and *M.sericea*), 5–10 mm long corolla tube (vs 24–55 mm long in *M.caliginosa* and 10–26 mm long in *M.sericea*), glabrous anthers (vs hairy in *M.caliginosa* and *M.sericea*) and the seed without papillate surfaces (papillate or canaliculate in *M.caliginosa* and *M.sericea*).

*Microchiritahairulii* is distinct from *M.viola* in the length of calyx lobes, 3–4 mm long, narrowly lanceolate (7–10 mm long, narrowly ovate in *M.viola*), corolla lobes very faintly striped or plain (conspicuous dark purple stripes in *M.viola*), having glandular hairs above the anthers, translucent pale brown (glandular hairs golden yellow, apically swollen in a cluster above the anther in *M.viola*)

#### Description.

Branched herb. **Stems** pale green or maroon green (in life), erect or sub-erect, if with a single leaf the stem elongated, flowering at *c.* 10 cm tall, shortly hairy, internodes 3.5–6.5 cm long. **Leaves** opposite, decussate, lowermost solitary; petiole pale green, 0.5–1.5 cm long, densely and shortly hairy; lamina ovate, 3.5–5.5(–9) × 2–2.5(–6.2) cm, yellowish green to dark green above, pale green to maroon beneath, thinly leathery (when fresh) or chartaceous (when dried), shortly hairy, base slightly cordate to cuneate, sometimes unequal, margin serrate, apex acute; midrib sunken above, prominent beneath, lateral veins 5–6 pairs, sparsely hairy, intercostal veins reticulate. **Inflorescence** epiphyllous, crested, 1–4-flowered, flowering from petiole base; bracts absent; pedicels green to maroon, to *c.* 1.2 cm long, glandular hairy. **Flowers**: calyx pale green, lobes almost divided to the base, 3–4 × 1–2 mm, acute, narrowly lanceolate, hairy outside, glabrous inside, margin entire; corolla tube very sparsely hairy outside, cream, 5–10 mm long, slightly curved, to *c.* 10 mm wide at the mouth, lobes very faintly striped, spreading, purple, throat cream, glandular hairs above the anthers, translucent pale brown, glistening; stamens 2, filaments whitish, inserted 6–8 mm from the base of the corolla, *c.* 4 mm long, straight, glabrous; anthers yellow, free or connate, without hairs, anther-thecae divergent, 1–2 mm long, staminodes 3, pale green, inserted *c.* 6 mm from the base of the corolla tube, 1.5–2 mm long; nectary pale yellow or cream, forming a complete ring, less than 1 mm high; pistil pale green, *c.* 1 cm long, ovary 2–4 mm long, *c.* 1.5 mm wide at the base narrowing to 0.5 mm below the stigma, shortly hairy, style *c.* 4 mm long, stigma deeply 2-lobed, *c.* 1 mm long, *c.* 2 mm wide, inserted between the anthers, with fine dense papillose hairs towards the tip; ovules cream, less than 1 mm long. **Capsules** green to maroon, 1.5–1.8 cm long, *c.* 2 mm wide, slender, densely hairy; calyx persistent, pale green or sometimes maroon, hairy. **Seeds** yellowish cream, many in one row, broadly ovate or elliptic, slightly elongated or rounded, surface without knobs or papillae.

#### Etymology.

The specific epithet honours Mohd. Hairul bin Mohd. Amin, a dedicated field collector who collected the species in the field.

#### Geographic distribution and ecology.

Endemic in Perlis, Peninsular Malaysia (Fig. 3). The species is restricted to karst limestone, where it grows on cliffs in crevices or soil pockets, or on a very thin soil layer at cave mouths, below the canopy or sometimes directly exposed to sunlight. It is found in very small populations.

#### Provisional regional conservation status.

Provisionally, the species is assessed as Endangered (EN B1ab(iii)). This endemic species occurs in five localities on the limestone hills. None of the hills lies in Totally Protected Areas, so the hills are potentially vulnerable to be exploited for other commercial uses (IUCN, 2012). Most of the hills are surrounded by paddy fields, rubber plantations and quarrying activities.

#### Specimens examined.

***Microchiritahairulii*** - PENINSULAR MALAYSIA: **Perlis.** Bkt. Jernih, February 2017, *Rafidah et al. FRI 86671* (KEP); Bkt. Keteri, November 2013, *Rafidah FRI 75880* (KEP); Bkt. Mata Ayer, February 2018, *Rafidah et al. FRI 90347* (KEP); Kg. Ujong Bukit, May 2017, *Rafidah et al. FRI 85902* (KEP).

***Microchiritacaliginosa*** - **PENINSULAR MALAYSIA: Kedah**: Kodiang, Bkt. Kaplu, 6 November 2009, *Rafidah FRI 64417* (KEP); *Ibid*., 27 May 2010, *Rafidah FRI 64545* (KEP). **Pahang**: Bkt. Charas, 15 October 1931, *Henderson 25233* (SING); *Ibid*., 26 November 1984, *Kiew RK 1557* (KEP); *Ibid*., 1 April 2008, *Rafidah FRI 55717* (KEP); Bkt. Chintamani, 4 October 1931, *Henderson SFN 25033* (SING, BK); Gn. Jebak Puyuh, 10 February 1986, *Kiew RK 2158* (KEP); *Ibid*., 10 February 1986, *Kiew RK s.n.* (KEP); Gn. Senyum, 30 July 1929, *Henderson s.n.* (SING); *Ibid*., 28 November 1984, Kiew RK *1587* (KEP); *Ibid*., 2 April 2008, *Rafidah FRI 55721* (KEP); Gua Bama, 3 April 2008, *Rafidah FRI 55726* (KEP); Gua Cermin R.F., 31 March 2008, *Rafidah FRI 55713* (KEP); *Ibid*., 31 March 2008, *Rafidah FRI 55714* (KEP); Gua Kechil, 30 July 2009, *Rafidah FRI 64379* (KEP); Kota Glanggi, 4 August 1929, *Henderson SFN 22419* (SING); *Ibid*., 2 April 2008, *Rafidah FRI 55724* (KEP); Panching, 26 November 1984, *Kiew RK 1571* (KEP); Panching F.R., 15 October 1931, *Henderson SFN 25223* (SING); Taman Negara, Batu Subuh, 5 October 1984, *Dawn RK 1470* (KEP); Taman Negara, Kuala Keniyam, 29 September 1982, *Kiew RK 1202* (KEP). **Perak**: Batu Kurau, December 1884, *Scortechini 1582* (SING); Ipoh, 17 July 1917, *Burkill 2558* (SING); *Ibid*., s.d., *Gordon GS 435* (KLU); *Ibid*., February 1904, *Ridley s.n.* (SING); Gopeng, 8 March1993, *Davison GD 4* (KEP); Gn. Lanno, 16 April 1925, *Mills 15061* (SING); Gn. Mesah, 20 April 1962, *Burtt B1665* (SING); *Ibid*., May 1902, *Curtis s.n.* (SING); Gn. Pipit, 23 April 1987, *Kiew RK 2524* (KEP); Gn. Rapat, 21 July 2009, *Rafidah FRI 64347* (KEP); *Ibid*., 9 March 1931, *Samsuri SA 560* (KLU, SING); Kinta, August 1898, *Curtis 3109* (SING); *Ibid*., January 1885, *King’s collector 7028* (SING); *Ibid*., 1885, *King’s collector 937* (SING); Kuala Dipang F.R., February 1890, *Curtis 2359* (SING); *Ibid*., 1898, *Ridley s.n.* (SING); Lenggong, Gua Badak, 28 October 2008, *Imin FRI 63212* (KEP); *Ibid*., July 1909, *Ridley s.n.* (SING); Sg. Siput, 21 May 1985, *Anthonysamy SA 842* (KEP); Sg. Siput Utara, 7 January 2015, *Rafidah FRI 82007* (KEP). **Selangor**: Batu Caves, 18 October 1983, *Anthonysamy SA 379* (KEP); *Ibid*., 19 November 1916, *Burkill 2253* (SING); *Ibid*., s.d., *Chung 331* (KLU), s.d., *Chung 390* (KLU); *Ibid*., February 1890, *Curtis 2359* (SING); *Ibid*., May 1902, *Curtis s.n.* (SING); *Ibid*., s.d., *Ding Hou 715* (KEP); *Ibid*., 23 January 1966, *Hardial 477* (SING); *Ibid*., 1889, *Kelsall s.n.* (SING); *Ibid*., 1 May 1981, *Kiew RK 1023* (KEP); *Ibid*., *Mohd. Kasim 391* (KLU); *Ibid*., 14 October 1966, *Ng FRI 1629* (KEP, SING); *Ibid*., 23 June 1889, *Ridley s.n.* (SING); *Ibid*., 4 November 1953, *Sinclair SFN 40066* (KEP, SING); *Ibid*., 29 November 1959, *Smith KEP 85205* (KEP); *Ibid*., s.d., *Yap SK 26* (KLU); Bkt. Anak Takun, 27 April 2006, *Phoon FRI* 5*1570* (KEP, SING); *Ibid*., 3 May 2005, *Sam FRI 50118* (KEP); Kanching F.R., 10 July 1995, *Julia JS 26* (KEP); Bkt. Takun, 3 November 1937, *Mohd. Nur 34389* (SING); *Ibid*., 9 March 1988, *Saw FRI 36215* (SING); *Ibid*., 20 November 1962, *Sinclair 10732* (SING); *Ibid*., November 1969, *Stone 8934* (KLU); *Ibid*., 21 September 1969, *Stone 8794* (SING); *Ibid*., 24 June 1933, *Symington FMS 30796* (KEP); *Ibid*., 8 May 1935, *Symington 39598* (KEP); Kanching, 16 March 1935, *Symington FMS 37431* (KEP). **Terengganu**: Taman Negara, Batu Biwa, 25 October 1986, *Kiew RK 2339* (KEP, SING); *Ibid*., 22 October 1986, *Kiew RK 2284* (SING).

***Microchiritasericea*** - **PENINSULAR MALAYSIA: Kedah**: Gn. Baling, 25 November 1941, *Corner s.n.* (SING). **Perak**: Gn. Rapat, 21 July 2009, *Rafidah FRI 64347* (KEP); *Ibid*., 26 May 2010, *Rafidah FRI 64544* (KEP); Gn. Tasek, Perak Tong Temple, 21 July 2009, *Rafidah FRI 64348* (KEP); *Ibid*., 23 October 1958, *Sinclair 9844* (SING); Ipoh, 4 July 1917, *Burkill 2552* (SING); *Ibid*., August 1898, *Corner s.n.* (KEP); *Ibid*., February 1904, *Ridley 11952* (SING); *Ibid*., February 1904, *Ridley s.n.* (KEP); *Ibid*., 18 August 1986, *Weber UPM 4167* (KEP); *Ibid*.,18 August 1986, *Weber s.n.* (KEP); Kuala Dipang F.R., 1898, *Ridley s.n.* (SING); Tambun, 10 September 1920, *Burkill 6284* (SING); Sg. Siput Utara, 29 January 2015, *Rafidah FRI 82017* (KEP).

***Microchiritaviola*** - **PENINSULAR MALAYSIA:Kedah**: Gn. Keriang, February 1890, *Allen s.n.* (SING); Langkawi, 20 November 1941, *Corner s.n.* (SING); Langkawi, Ayer Hangat, *Chung 505* (KLU); Langkawi, Bkt. Malut F.R., 4 November 1968, *Keng 80* (SING); Langkawi, Batu Puteh, August 1941, *Nauen 38120* (SING); Langkawi, Kuah, 8 November 1968, *Chung RC 7* (KEP); *Ibid*., 5 November 2009, *Rafidah FRI 64407* (KEP); *Ibid*., 1979, *Stone 14349* (KLU); Langkawi, P. Langgun, 4 November 2009, *Rafidah FRI 64398* (KEP); Langkawi, P. Timun, 1926, *Holttum 17433* (SING); Langkawi, Selat Panchor F.R., 19 November 1941, *Corner 37832* (SING); *Ibid*., 21 November 1934, *Henderson SFN 28931* (SING); *Ibid*., November 1934, *Henderson SFN 29185* (SING); *Ibid*., 3 November 2009, *Rafidah FRI 64388* (KEP); Langkawi, Tg. Rhu, 21 November 1993, *Anthonysamy SA 1144* (KEP, SING); Langkawi, Tg. Sawah, 22 November 1941, *Corner s.n.* (SING); Langkawi, Tg. Terai, 13 November 1941, *Corner s.n.* (SING).

## Supplementary Material

XML Treatment for Microchirita hairulii
